# Learning From the Adoption of a Readmission Clinical Decision Support Tool: Group Model Building Approach

**DOI:** 10.2196/87522

**Published:** 2026-04-29

**Authors:** Nina Rachel Sperber, Sarah Elizabeth Haas, Jiaxin Gao, Samantha Hamelsky, Theresa Kiki-Teboum, Afraaz Malick, Rishab Pulugurta, Jacqueline Rodriguez, Hana Shafique, Eden Singh, Kriti Vasudevan, Scott Rockart, David Gallagher, Adam Johnson

**Affiliations:** 1 Department of Population Health Sciences, Duke University School of Medicine, Box 90046, Durham, NC, 27708, United States, 1 919-672-2280; 2 Durham VA Health Care System, Durham, NC, United States; 3 Trinity College of Arts & Sciences, Duke University, Durham, NC, United States; 4 Duke University School of Medicine, Durham, NC, United States; 5 The Fuqua School of Business, Duke University, Durham, NC, United States; 6 Department of Medicine, Duke University School of Medicine, Durham, NC, United States; 7 Division of Vascular and Endovascular Surgery, Department of Surgery, Duke University School of Medicine, Durham, NC, United States

**Keywords:** clinical informatics, implementation science, risk prediction, clinical decision support, system science

## Abstract

**Background:**

Computerized clinical decision support (CDS) has the potential to improve patient outcomes by offering evidence-based guidance at the point of care—enhancing guideline adherence and diagnostic accuracy—and supporting system-level outcomes by enabling predictive analytics for more efficient resource planning. Prior work has identified factors that affect adoption, such as clinicians’ expectations of usefulness, ease of use, alignment with workflows, and resources to support utilization. However, CDS adoption is not static and changes according to dynamic systems of behaviors and workflows, requiring a deeper understanding of how evolving conditions affect implementation and outcomes.

**Objective:**

To explore the dynamic factors influencing CDS adoption, we examined the implementation of the “Unplanned readmission model version 1,” developed by Epic Medical Records System, at Duke University Health System, using group model building and system dynamics modeling.

**Methods:**

We first conducted group model-building workshops with staff (case managers, physical and occupational therapists, hospitalist faculty physicians, and resident physicians) who participate in decisions about discharging patients. Study team members guided participants to identify and connect variables in causal loop diagrams. We coded workshop transcripts in software designed for system dynamics analysis to identify themes, aggregated them into a causal loop diagram, and reviewed them with participants to converge on a common model. A team member applied equations to the pathways and tested data to simulate conditions leading to full, limited, or no adoption of a tool.

**Results:**

We identified key balancing loops driven by external pressure (eg, Centers for Medicare & Medicaid Services penalties) that motivated initial adoption and reinforcing loops based on perceived internal benefits to sustain use. While institutional incentives led to early training and tool use, efforts declined due to staff turnover, competing priorities (eg, COVID-19), and workflow changes. Reinforcing loops emerged when staff described clinical utility, such as improved discharge planning and team communication. However, staff also suggested that these loops were often weak due to difficulty linking the use of the tool to outcomes in real time. Simulation modeling showed that while strong external pressure and rapid training led to initial success, interest in using the tool waned as workflows improved and readmission rates approached Centers for Medicare & Medicaid Services goals. When conflicting priorities were introduced, adoption stalled earlier, and fewer staff were trained. In contrast, when internal motivation was strengthened by reducing the amount of evidence needed to perceive success, individual interest remained high even as institutional attention declined, sustaining tool use and further reducing readmissions.

**Conclusions:**

External pressure to improve can be a strong motivator for initial adoption, but in the face of conflicting demands for attention, it may fall short of sustained long-term tool use. Tools are more likely to have extensive and sustained use when those using the tools can perceive internal benefits.

## Introduction

Computerized clinical decision support (CDS) tools, health IT systems that deliver patient-specific recommendations during clinical workflows, have the potential to transform health care delivery. By delivering evidence-based guidance at the point of care, these tools enhance clinical decision-making, improve patient outcomes, and enable health care organizations to anticipate future resource needs through predictive analytics, leading to more efficient planning and resource allocation [1-4]. A targeted literature review published in the *Interactive Journal of Medical Research* found that CDS systems positively impacted quality assurance in 69% of the studies analyzed and provided clinical benefits in 41% compared to usual care [5]. Quality assurance was supported through improved adherence to clinical guidelines, better diagnostic support, monitoring practices, and improved screening and treatments. Clinical benefits included improved diagnostic accuracy, clinical decision-making, and treatment selection. The effectiveness of CDS tools is closely linked to their successful adoption at the bedside.

Understanding the factors that influence clinicians’ adoption of CDS tools is essential for designing effective implementation strategies. The Unified Theory of Acceptance and Use of Technology, developed in 2003, has identified 4 key concepts that could influence new information technology adoption: performance expectancy (belief that using the system will help one improve job performance), effort expectancy (ease associated with the system including individual perceptions and design features), social influence (perceived social pressure to use the system), and facilitating conditions (perceived organizational and technical support for system use) [6]. Lui et al [7] found that performance expectancy and effort expectancy were significant predictors of CDS adoption and that these beliefs depended on individuals having autonomy and agency. A meta-analysis that examined predictors of health care practitioners’ intention to use artificial intelligence–enabled CDS through the lens of the Unified Theory of Acceptance and Use of Technology found that facilitating conditions influenced performance and effort expectancy and, in turn, intention to use artificial intelligence–enabled CDS [8]. A recent study by Wang et al [9] additionally demonstrated the importance of top management support, in which senior administrators recognize the importance of the information system and are actively engaged in its implementation, for adoption by positively shaping not only performance and effort expectancies but also social influence. Other work has shown that clinicians are more likely to adopt tools that they view as accurate and useful when these tools integrate with existing workflows and have resources available to act on the risk scores [10,11]. Conversely, tools perceived as irrelevant and interruptive to workflows have led to “alert fatigue” and low utilization rates [12,13]. While this work sheds light on how multilevel factors affect CDS adoption, there is additionally a need to understand the dynamics of the systems within which CDS functions, with behaviors and workflows that change over time; this information is important for developing strategies to sustain adoption [14].

Systems-aligned precision medicine describes CDS strategies that account for complex and dynamic systems of care, not only for the patient but also for the clinician engaged in decision-making [15]. To understand the dynamic complexity of factors that affect adoption, we developed a study to explore facilitators and barriers in the adoption of a single use case, the implementation of “Unplanned readmission model version 1,” developed by Epic Medical Records System, at Duke University Health System using system dynamics modeling [16]. Readmissions have been identified as an essential quality indicator and linked to institutional financial incentives through the Centers for Medicare & Medicaid Services (CMS) Hospital Readmission Reduction Program [17,18]. Due to the significant impact on patients and institutional stakeholders, many readmission risk scores have been developed to target high-risk patients with an improved discharge process [19,20]. We engaged interest holders in developing a system dynamics model by conducting group-based modeling sessions with health system staff actively involved in the discharge process. Through these sessions, we aimed to uncover factors that inhibit or promote the use of the readmission risk score and identify potential solutions to enhance its adoption and effectiveness. After completing the workshops, we built a mathematical version of the model to explore the logic captured in the causal loop diagram. This type of model, known as a simulation model, provides an opportunity to determine if the loops can create the behaviors reported in the workshops and under what conditions they may create other, not yet observed, behaviors.

## Methods

### Ethical Considerations

The protocol was reviewed by the Duke Health institutional review board and determined to be exempt from further institutional review board review (Pro00118266). The exemption was granted under category 2, covering minimal‑risk research involving survey or interview procedures, and was consistent with institutional policy and applicable federal regulations. Because the study was deemed exempt, written informed consent was not required; participants were provided with study information and informed that participation was voluntary. No direct identifiers were collected, and the data were managed in accordance with Duke Health policies on privacy and confidentiality. Participants did not receive compensation for participation.

### Setting

In 2017, Duke Health implemented Epic’s “Unplanned readmission model version 1” (hereafter referred to as the Epic readmission risk score) in response to the need to reduce unplanned readmissions. CMS considers readmissions a key quality indicator and thus implemented the Hospital Readmissions Reduction Program to incentivize institutions to reduce their readmission rates [18]. Duke University Hospital (DUH) is a quaternary academic hospital located in Durham, North Carolina, providing high-acuity care across specialties. The hospital serves a diverse patient population from Durham County, Wake County, and surrounding areas in North Carolina.

**Figure 1.**
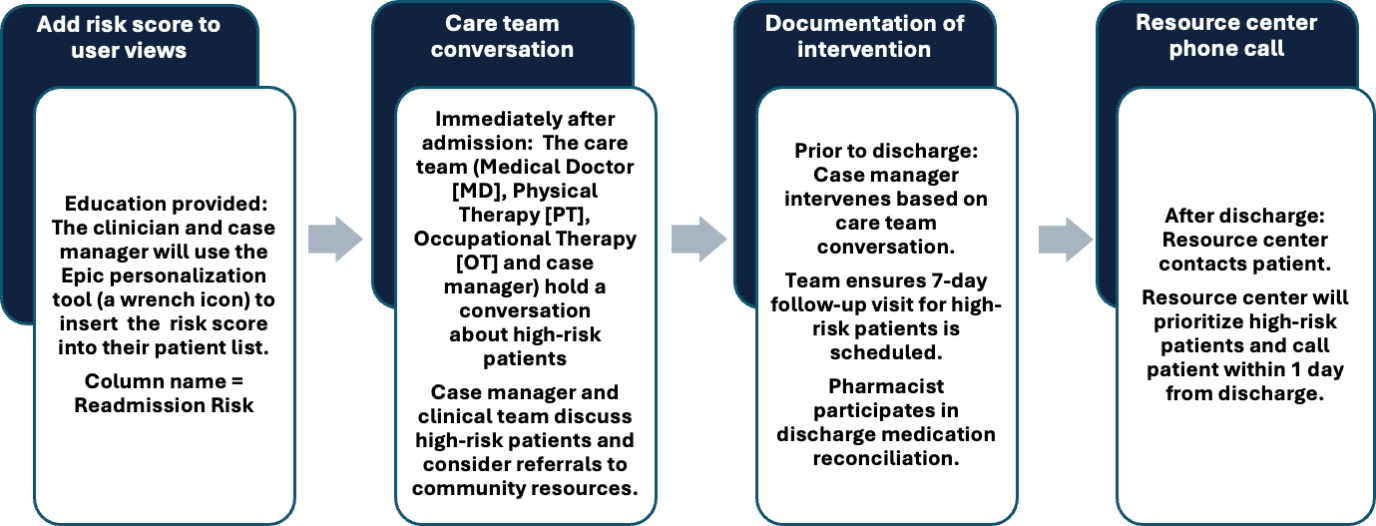
Integration of the Epic readmission risk score into clinical workflows used during training and the initial implementation.

At a hospital like DUH, with almost 1200 beds and an average of more than 100 discharges a day, it would be impossible and unnecessary to apply all discharge resources to every patient. Hospitals like DUH need to stratify their time and resources based on patients who have the highest risk of being readmitted. The Epic readmission risk score was introduced at DUH in November 2017 to proactively identify patients at high risk of readmission and implement targeted interventions to reduce readmission rates [16]. This CDS tool analyzes Epic’s electronic health record data to generate a readmission risk score, ranging from 0 to 100, along with corresponding color codes: red for high risk, yellow for medium risk, and green for low risk. Predictors such as patient age, laboratory variables, clinical diagnosis variables, medication numbers and classes, order types, and utilization variables are used to populate the score. This score and color code are then displayed within the patient’s electronic health record, allowing clinicians to make informed decisions about postdischarge care and follow-up. Figure 1 shows the proposed integration into clinical workflows with multidisciplinary review at discharge and the implementation of close follow-up and additional services upon discharge.

### Project Design

**Figure 2.**
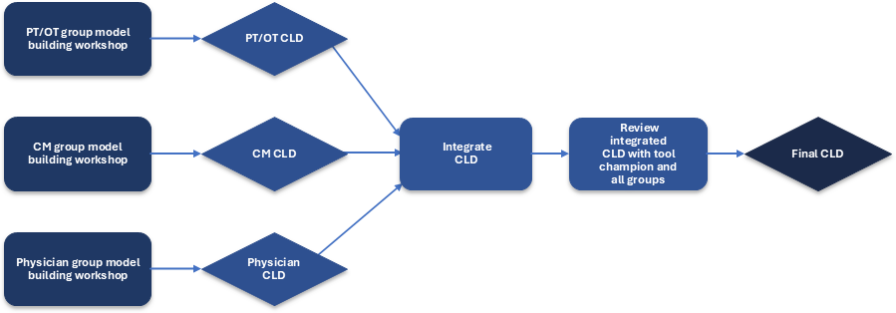
Study diagram. PT/OT: physical therapy/occupational therapy, CM: case manager, CLD: causal loop diagram.

To explore adoption in this setting, we used group model building, a process that engages stakeholders in developing a system dynamics model. We performed 3 group model-building workshops with small groups of staff involved in the discharge process at DUH. These staff included case managers, physical and occupational therapists, and internal medicine and general surgery resident physicians. The workshops aimed to understand the factors influencing the uptake and sustained use of the Epic readmission risk score. In each session, the team facilitated the production of a causal loop diagram meant to represent the utilization of the Epic readmission risk score at the hospital, from the participants’ perspectives. A final review session was held with members from all groups, as well as the chief medical officer, who initially led the implementation of the tool, and faculty hospitalist physicians. Figure 2 provides a visual representation of how our causal loop diagram was developed by the workshop participants and facilitators.

Before the sessions, a script was created detailing the intended agenda of the workshops. The team adopted a process for variable elicitation and causal loop diagramming from *Scriptapedia*, a handbook of scripts for developing structured group model-building sessions [21]. In this structure of group modeling, there is a core modeling team that includes facilitators who lead the discussion, a recorder who monitors the audio recording while documenting important themes, and a “wall builder” who draws content on a whiteboard [22]. In these workshops, the facilitators (primary and secondary) first introduced themselves, the problem of declining use of the risk score, and the objective of constructing a causal loop diagram. The facilitators then prompted participants to describe their roles in the discharge process and discuss their experiences, or lack thereof, with the tool. The facilitators invited participants to contribute, by speaking or writing, suggestions about conditions that have affected tool use and worked with the group to identify variables and feedback loops. While the “wall builder” recorded the variables and diagram on a whiteboard, participants additionally had the opportunity to “take the pen” and contribute to the diagram directly. These workshops were transcribed, and the transcriptions were then coded in the DynamicVu (DynamicVu) software for key variables and causal links to match quotes from participants to parts of the causal loop diagram [23]. The study team worked together to integrate data from the 3 workshops into a single causal loop diagram, which was subsequently converted into a mathematical simulation model to assess internal validity, utilizing Vensim PLE (Ventana Systems, Inc). A final review session was conducted with a few members of each group, along with the chief medical officer at DUH, who led the initial implementation of the tool in 2017.

## Results

### Overview

**Figure 3.**
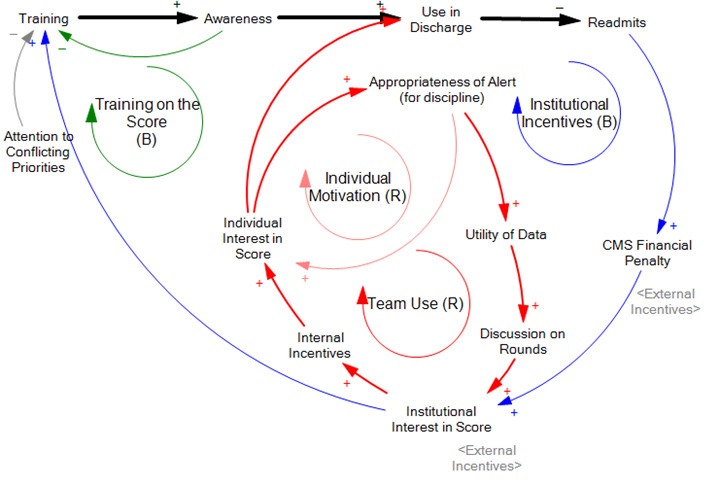
Simplified causal loop diagram developed from the workshops directly. CMS: Centers for Medicare & Medicaid Services.

We successfully completed the 3 interest holder workshops with the active participation of 5 to 10 clinicians per workshop. We then held a final group workshop with representatives from each group with active participation. As we explored one tool, we developed a set of dynamic hypotheses based on workshop participants’ insights. The analysis revealed 3 key themes that explain the dynamics observed in the organization’s efforts to lower the readmission rate. The three themes included institutional-level learning and reinforcement, individual-level learning and reinforcement, and the training and support of a champion (Figure 3).

### Organizational Response to Lower Readmission Rates

The first feedback loop identified was the organization’s response to the external desire to lower the readmission rate (“Institutional Incentives” loop). This initiative to implement the Epic unplanned readmission score, driven by the institution’s general concern for reducing readmissions, began prior to these modeling workshops. The tool was initially implemented through a pilot project that included an evaluation component led by a multidisciplinary team comprising members from hospital medicine, case management, clinical operations, biostatistics, and informatics, who encouraged training programs on the tool [16]. The hope was that this training would lead to increased use and thus better discharge plans for patients with a high risk of 30-day readmission. The initial push for tool use led to more training and awareness.

Our workshops led us to believe that while some staff still use the tool, many are unaware of its presence or capabilities, with usage generally declining over time rather than becoming widespread. We explored with staff potential explanations for the lack of widespread awareness and use of the tool, resulting in a balancing feedback loop, in which initial training that aimed at equipping staff with the necessary skills to achieve the desired outcomes dropped off, and other circumstances distracted attention away from continued training and use. We identified several reasons why training may have declined. These included normal turnover and events such as the COVID-19 pandemic, which placed other constraints on training. Additional changes, including arguably positive ones, changed the workflow by incorporating alternative practices and tools that may have been inspired by and substituted for the tool. The case managers described how the pilot program to introduce the score had changed their behavior by providing a visual cue to collect more information for specific patients indicated as “high risk” for readmission with a red alert. Some participants suggested there would be increased adoption if factors included in the algorithm aligned with their conceptual model of risk in these patients. Over time, however, they internalized the criteria associated with the alert, and those characteristics themselves became the cues. For example, one case manager said:

*I think that the … [alert] started out like great in the beginning because it was the startup of a new system. So, it was like, hey, this is your visual to know that we have to do something. And maybe now we lost part of that visual because now we know what we’re looking for. Practice had changed since the introduction of the score to collect additional information for patients flagged as “high risk” for readmission. … it just became part of our workflow of knowing in our assessment, like of room to assess more in depth, right, if that makes sense. It gave us the tools to then ask more in-depth questions, but then that became part of our assessment process.*

This organizational-level balancing loop indicates that, over time, as the readmission rate reaches a certain threshold, the institutional interest, driven by external pressure from the CMS penalty, will eventually drop [18]. The level of institutional interest, in turn, would affect individual staff members’ interest in using the score. As one resident said, “… if the institution is saying we need to reduce readmissions at the end, I’m going to do everything I can to reduce readmissions ...”

### Reinforcement Through Perceived Benefits

Over the long term, however, as the initiative transitions from the initial training phase to broader institutionalization, the efforts of these champions may be partly or wholly replaced by individual or organizational learning processes that reinforce tool use (see “Individual Motivation” and “Team Use” loops, which are reinforcing). These feedback loops emerged from the hope that staff would recognize the benefits of the training and the resulting improvements in readmission rates.

 At both the individual and team levels, reinforcement can come from staff using the tools, observing the tool’s appropriateness for their work, and becoming more committed to its use. Clinicians discussed how evidence of the effectiveness of the tool for their practice is an important driver for adoption. They indicated that they encounter many scores in their field and said that those they want to use “have been shown to be effective” and “provide obvious, clear direct value.” In this case, they want to know what contributes to the score and “if any of that relates back to discharge.” One resident explained:

*It’s on par with a new risk score where we don’t actually know- like you’re telling us that this is going to work or this is going to do this, but we have no backing or no evidence of that. So, I think that’s another aspect to consider is like, yeah, we could look at it, but we don’t actually know how much this makes a difference, how impactful this truly is.*

Staff said that they would value the data generated from the score if they contained information that would affect their decisions in real time. One physician said:

*The only scores that we have time to spend on in medicine when we’re running around 8000 times a day are the ones that directly affect our decision-making in that exact moment.*

They would then incorporate the information into the discussion during multidisciplinary rounds, which aligns with institutional interests. One case manager described a push for bringing multidisciplinary teams together at the same time as the introduction of the readmission risk tool:

*There’s been a push for pulling the multidisciplinary team together to improve communication. So, there’s been lots of work all happening at the same time, and then we’ve got the hospital care hub ... part of the interdisciplinary ... case reviews and rounds.* 

These reinforcing loops were based on the expectation that visible positive outcomes would motivate continued engagement and institutionalization of the practices introduced during the training. A medical doctor said:

*If the institution is saying we need to reduce readmissions at the end, I’m going to do everything I can to reduce readmissions if I’m incentivized to do so … If using this helps, then I will keep using. So then that gets into clinical utility.*

A physical therapist suggested a similar sentiment, saying that their use would depend on evidence that the score had clinical utility for the kinds of factors that they assess to prevent discharge. For example, “I think it would be helpful if we knew a little bit more about … what’s contributing to the score and if there’s any … factors … that relate back to discharge ...”

### Risk for Reinforcement

We additionally recognized areas of risk that could prevent this reinforcement from taking over. In our setting, the reinforcing loops could be weak and thus unable to sustain the program at a high level. The “Individual Motivation” loop may be weak because readmission is a rare event and often not directly managed by the discharging team, so team members are unlikely to see the immediate impact of their actions by observing fewer readmissions. In addition, the clear causal connection between the recommended interventions and the intended outcome may be difficult to observe in real time. On the other hand, length of stay was much more directly observed by discharging clinicians, and efforts to reduce length of stay may come into conflict with efforts to reduce readmission, which can at times increase length of stay. Therefore, the natural reinforcing loops are weak, and external reinforcement through training and awareness campaigns is required. However, the ongoing work of champions and reinforcement can be disrupted in various ways, leading to a decline in tool awareness and use over time. As people change roles and take on new tasks, the ongoing active support of champions is likely to diminish, leading to a drop in training and awareness, especially as other changes crowd out efforts to support the use of the tool. Reinforcement relies on individuals being able to observe the tool’s value in their work or, at higher administrative levels, recognizing the value of the tool and creating incentives. One medical doctor described how awareness of a tool would decline over time if it was perceived as inappropriate for their workflow and not easily integrated into the work of new team members:

... *that institutional knowledge you have to maintain over those cycles. And that means that it has to be, like directly applicable, because I’m helping set up my interns who will then help set up someone for another four years, right. So, if like, if I don’t know about it now, then no one... [will] four years from now.*

Additionally, participants expressed difficulty in observing the tool’s value at an individual level for low-probability events (readmission is a relatively low probability event, making it harder to see the tool’s signal). Resistance to broader changes in the workflow for how decisions are made could also impede the use of the tool. For example, participants observed barriers as the institution encouraged moving to a more consultative, multidisciplinary group process. As one medical doctor said:

*I think one thing is hierarchical decision-making is a barrier in the sense that like if the case managers or the residents are the target utilizers for the tool, but we’re not the primary stakeholders in decision-making, then it’s … kind of futile like I, I guess to make it very concrete. It may be difficult for individuals or organizations to recognize the value of the tools, and it may be challenging to find ways to ensure continued use.*

### Internal Validity Test With Simulation Modeling

**Figure 4.**
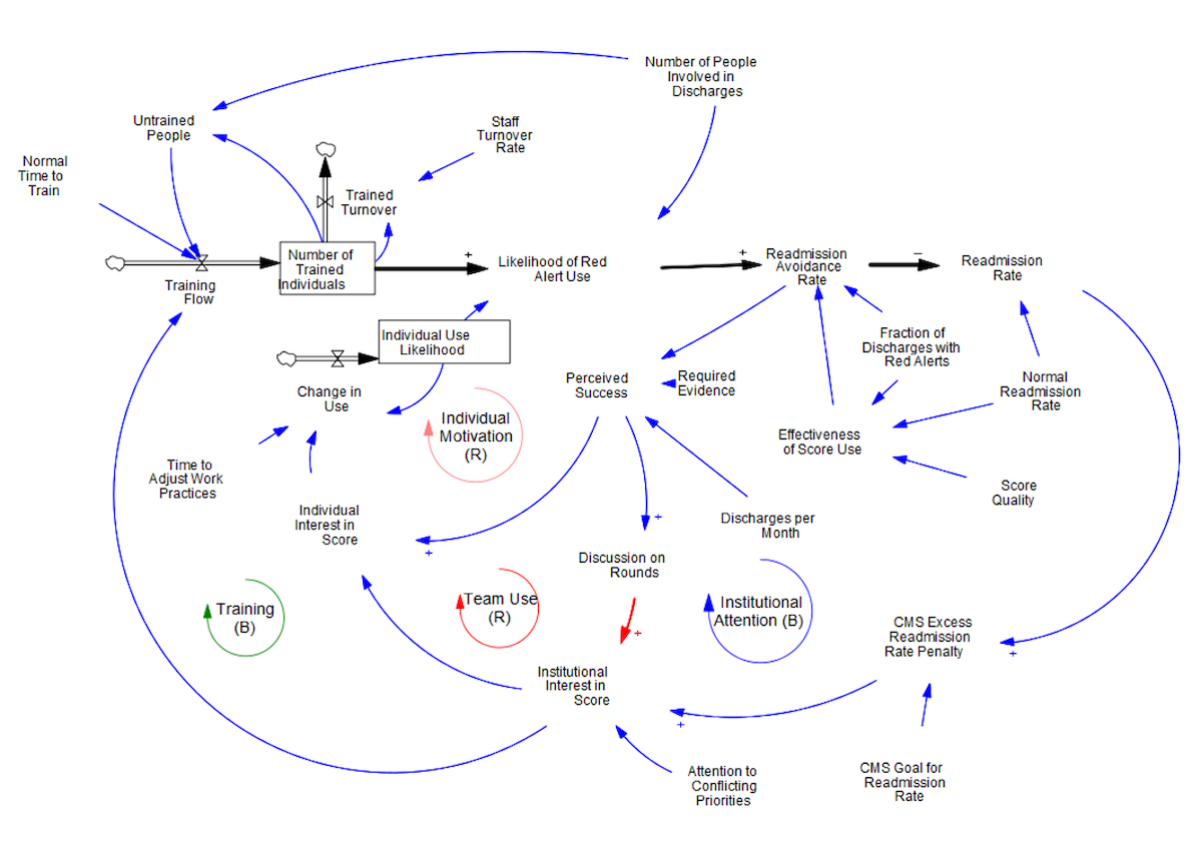
Simulation model derived from the causal loop diagram. CMS: Centers for Medicare & Medicaid Services.

A mathematical simulation model was developed from the initial causal relationships (Figure 4; see Multimedia Appendix 1 for equations). The primary objective in building the mathematical model was to adhere as closely as possible to the causal-loop diagram. To that end, the equations leverage the simplest possible “accounting” or probabilistic logic wherever feasible. As an example of an “accounting” logic, “Awareness” is modeled as the “Number of Trained Individuals,” with that number increasing when people are trained and decreasing when trained staff depart. As an example of probabilistic logic, the “Likelihood of Red Alert Use” is modeled as the fraction of people involved in discharges who are trained (the Number of Trained Individuals divided by the Number of People Involved in Discharges), multiplied by the fraction of discharges during which trained individuals pay attention to the score. The product of these 2 variables should capture the fraction of people involved in each discharge who are both trained to use the score and are actively looking at the score.

The “Readmission Avoidance Rate” is central to the model, which captures the reduction in readmissions. This equation leverages probabilistic logic. The “Readmission Avoidance Rate” is the product of 3 parameters: the “Likelihood of Red Alert Use,” the “Fraction of Discharges with Red Alerts,” and the “Effectiveness of Score Use.” In the best possible case, 3 factors are true. First, every red alert is given full attention (the Likelihood of Red Alert Use equals 1). Second, everyone and only those who would later be readmitted are given a red alert (the Fraction of Discharges with Red Alerts equals the Normal Discharge Rate with no false positives). Third, attention to the score leads to interventions that are entirely effective in avoiding readmission (the Effectiveness of Score Use equals 1). If these conditions are true, the “Readmission Avoidance Rate” will equal the “Normal Readmission Rate,” and readmissions will drop to zero.

Understanding this logic around avoiding readmissions takes us most of the way to understanding how the model captures workshop themes of balancing institutional incentives and training on the score loops and the 2 reinforcing loops of individual interest and team use. As derived from the workshop discussions, the 2 institutional level loops are strong initially, when readmissions are high enough to drive institutional interest in the score, leading to training and individual attention to the score. In the simulation model, the training loop is a balancing loop because if training is successful, the use of the tool will lead to lower readmissions and thus the penalty to fall, resulting in a drop in institutional interest and, with it, training. The simulation model similarly recognizes that a reduction in the financial penalty reduces institutional interest in and attention to the score with a similar logic, except that, rather than the reduced readmission rate leading to a fall in training, it leads to a fall in individual interest in the score.

We grounded the connection between the readmission rate and institutional interest in the score using the CMS “Excess Readmission Rate Penalty.” When institutional readmission rates exceed the CMS goal, CMS payments to institutions decrease, driving institutional interest in tools (in this case the score) that can lower the rate to below the CMS goal for the Readmission Rate. For simplicity, we have assumed that this penalty depends on the fractional amount by which the institution’s readmission rate exceeds the CMS goal (This is both a simplified calculation of the CMS penalty and a change in wording from the CMS original word “score” to “penalty” to avoid using the word “score” for multiple different ideas in the model.).

**Figure 5.**
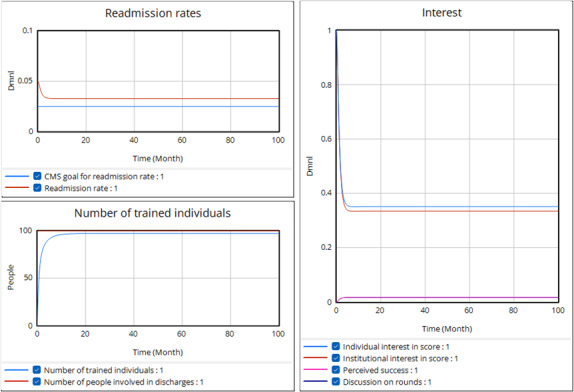
Simulation run with strong balancing loops. The equations and parameters for this run are given in Multimedia Appendix 1. CMS: Centers for Medicare & Medicaid Services.

We can see the behavior of these 2 balancing loops in a run of the model (Figure 5) set, so that these are strong loops, and the other (reinforcing) loops are weak. Here, training is quick (all untrained people are trained within 1 month), and turnover is low (only 1% per month), so the number of qualified individuals can rise quickly and, if so, stay high. Red alerts are set to match the regular readmission rate with no false positives, so attention to the score always leads to avoiding readmission. In addition, we have assumed that the institution has little need to address conflicting priorities. With this favorable set of parameters, the high initial readmission rate relative to the CMS goal leads to high institutional interest that drives training and individual interest in the score. With trained people using the score regularly, the readmission rate falls rapidly. The number of qualified individuals never quite rises to equal the number of individuals involved in discharges because of the delay in training as staff depart.

Additionally, institutional and individual interest in the score decreases as the readmission rate approaches the CMS goal. While we see a highly successful introduction of the tools with widespread and effective use, external pressure can only drive the effort so far. As the institution gets better, we can expect balancing loops to stop short of complete success because the organization relies on some continued external pressure (provided by high readmission rates) to sustain interest.

**Figure 6.**
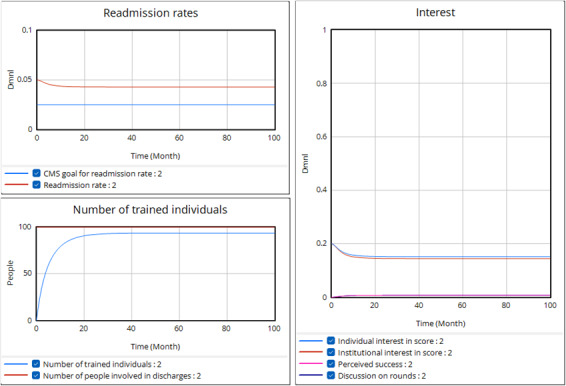
Simulation run with greater attention to conflicting priorities weakening the balancing loops. In this run, the Attention to Conflicting Priorities has been raised from 1 to 5 with no other changes to the model equations or parameters from the previous run. CMS: Centers for Medicare & Medicaid Services.

As is likely apparent from the discussion above, many factors could limit the effectiveness of these 2 balancing loops. A second run (Figure 6) shows one of these factors affecting both loops. Imagine a scenario where, as hospital staff discussed, the institution faces substantially higher conflicting priorities such as a pandemic or a stronger focus on reducing length of stay. These alternative priorities, which may have a more proximal impact than hospital readmissions, could reasonably divert attention and resources from training on the readmission risk score, thereby increasing the gap between the number of trained individuals and the number of people involved in discharge. While the readmission rate decreases as a result of the initial push for training and education, institutional interest will drop sooner with conflicting priorities, leading to reduced training and diminished individual interest in the score. Although still successful, the program results in fewer people being trained and a smaller reduction in readmissions. The shortfall is even more significant because the organization relies on continued pressure (provided by even higher readmission rates) to maintain interest.

**Figure 7.**
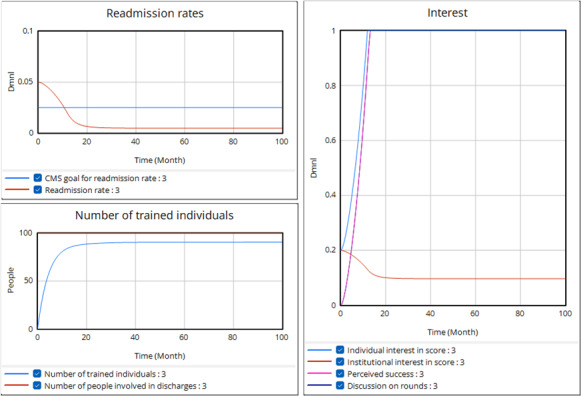
Run with reduced required evidence strengthening the reinforcing loops. In this run, the Required Evidence has been reduced from 100 persons/month to 3.5 persons/month with no other changes to the model equations or parameters from the previous run. CMS: Centers for Medicare & Medicaid Services.

Having set the parameters (notably raising attention to conflicting priorities) so that the balancing loops alone lead to limited success, we then explore conditions under which the reinforcing loops could take over and drive more significant use of the tool and improvement in the readmission rate (Figure 7). The main change in this run is to reduce the Required Evidence, which refers to how many discharged patients each month must avoid readmission for individuals and groups involved in discharges to perceive tool success. We were mainly trying to capture how evident it is that unplanned readmissions were avoided, as we heard in the group model building (GMB) sessions. In this case, the readmission rate falls below the CMS goal, causing a drop in institutional interest. Despite the loss of external pressure from the CMS, the program continues to be driven by the excitement generated internally from perceived tool success. Institutional interest is even lower than before, and thus so is training, though it is buoyed by internal pressure from use on rounds. Still, individual interest is far higher, which offsets the drop in training.

## Discussion

### Principal Findings

The causal loop diagram represents hypotheses that emerged from workshops regarding the adoption of a readmission risk prediction tool. The analysis of these feedback loops provided insights into the organizational dynamics influencing the success and sustainability of the initiative to reduce readmission rates. The findings inspired the development of a mathematical model to explore the logic of these loops in greater depth.

The balancing loops identified during the workshops highlighted challenges in maintaining the momentum of training and the need for ongoing reinforcement, both “facilitating conditions,” to achieve the desired reduction in readmission rates [6]. Eagerness to reduce readmission rates to meet an external goal can only take adoption so far. Although feedback loops tied to external goals are intended to close the gap between current readmission rates and benchmark targets, the narrowing of that gap can paradoxically reduce effort. This effort refers to the ongoing commitment of time, training, and behavioral adaptation required for successful tool integration into clinical workflows. Depending on how quickly the effort to close the gap diminishes, as it might do rapidly in the face of strong external pressure for attention elsewhere, effort may fall too low before the gap is closed, and the tool may be used far less than its potential.

For sustained success, we believe the implementation of the readmission risk score requires additional input. The workshops provided indications that these additional inputs, in the form of reinforcing loops, would likely come from individuals across several disciplines perceiving internal benefits from the tool. Similarly, Venkatesh et al [6] suggested that while institutional influence, such as the perception that management wants staff to use the tool, tends to motivate new technology use in the early stages of implementation, over time, employees will rely less on these external drivers as they gain experience and integrate it with their workflow. If these internal reinforcing loops are strong, the process is likely to sustain itself without additional training or input from the tool developer. However, there is a risk of relying too heavily on the education of clinical team members and getting them to incorporate it into their own workflow. If the reinforcing loops are not strong, there are implementation strategies that could provide additional input. For example, a monitoring and evaluation system could function as an integral control mechanism for the Institutional Attention balancing loop that maintains tool use even when performance improves by reminding people of the improvements that could be lost without continued efforts [24,25]. Interventions might include a champion who could respond to feedback by adjusting support, such as by reinforcing the Training loop, to reduce long-term drift from desired tool use or an electronic health record–integrated dashboard that provides immediate feedback on key metrics to address the need for evidence about the CDS within the Individual Motivation and Team Use loops [26,27]. The key lesson then is that widespread and sustained adoption is far more likely if those who use the tool are able to see a positive impact in daily practice on patient care decisions and outcomes.

 By using a group model-building approach, we uncovered how these constructs interact over time through feedback loops at our institution. Our simulation model showed that while external pressure can drive initial adoption, it often leads to balancing loops that limit long-term success. Competing priorities that arise only magnify the effect of these balancing loops by directing attention away from initiatives to promote use. In contrast, reinforcing loops—driven by perceived internal benefits and team-level learning—can sustain tool use even after institutional interest declines. However, these reinforcing loops are fragile and require conditions such as visible tool impact, low required evidence thresholds, and integration into daily workflows to take hold. This dynamic systems perspective highlights the importance of designing implementation strategies that not only address user perceptions but also anticipate and support the evolving organizational and social conditions in which CDS tools are used. Modeling system behavior with different types of CDS tools and across diverse institutional contexts will help build a more generalizable theory of how system structure shapes adoption. For example, tools that provide more immediate feedback, such as real-time alerts for clinical deterioration, may be less vulnerable to competing priorities than tools with delayed feedback. To advance this work, it will also be important to build protocols to measure the effectiveness of the GMB and derived solutions: this could include measuring changes in staff understanding of the problem as a result of participating in the GMB and changes in implementation outcomes as a result of solutions implemented based on GMB insights. While this model explores the dynamics of implementing one type of CDS in one institutional setting, it provides a foundation for future research to extend toward a broader systems-aligned precision medicine framework.

### Conclusions

We have developed a system dynamics model to approximate the driving and impeding forces related to the adoption of a readmission risk tool at a quaternary academic hospital through group model-building approaches. While we examined one specific outcome of CDS tool use, where there remains uncertainty about the widespread adoption of the tool into clinical workflow, the workshops and modeling point to the potential for a range of different outcomes in different settings. The decline in adoption of this specific score likely relates to the rare nature of the event, as there is limited real-world experience of the impact of the risk model on day-to-day experiences, combined with a decrease in active training and promotion due to competing priorities with the COVID-19 pandemic and an additional focus on length of stay. Understanding the implementation of these risk algorithms in a dynamic system will help developers iterate and improve implementation moving forward. Further research is planned to test the model predictions against a wider range of cases and settings, calibrate the model to test for external validity of the arguments, and refine the lessons.

## Supplementary material

10.2196/87522 Multimedia Appendix 1 Equations used in the simulation model.

## Abbreviations

CDS: clinical decision support
CMS: Centers for Medicare & Medicaid Services
DUH: Duke University Hospital
GMB: group model building
