# Supplementary material for: Learning From the Adoption of a Readmission Clinical Decision Support Tool: Group Model Building Approach
Source: JMIR Hum Factors. 2026 Apr 29;13:e87522. doi: 10.2196/87522 (PMC13128158; doi:10.2196/87522)
Supplement: Multimedia Appendix 1 [file humanfactors-v13-e87522-s001.docx]

## Model Equations

(01) Attention to Conflicting Priorities= 1

Units: Dmnl [1,10]

(02) Change in Use= (Individual Interest in Score - Individual Use Likelihood) / Time to Adjust Work Practices

Units: Dmnl/Month

(03) CMS Excess Readmission Rate Penalty= (Readmission Rate - CMS Goal for Readmission Rate ) / CMS Goal for Readmission Rate

Units: Dmnl

(04) CMS Goal for Readmission Rate= 0.025

Units: Dmnl [0,0.2]

(05) Discharges per Month= 100

Units: People/Month [0,200]

(06) Discussion on Rounds = Perceived Success

Units: Dmnl

(07) Effectiveness of Score Use= Score Quality * MIN( 1, (Normal Readmission Rate / Fraction of Discharges with Red Alerts))

Units: Dmnl [0,1]

This says how often a readmission will be avoided when the score is used for a red alerted patient. We assume no type I error (all red alert patients are patients will be readmitted if not prioritized for care) up to the point where red alerts become more common than readmission (above which additional red alerts represent type II error, alerts for patients who are not going to be readmitted) thus the effectiveness must fall to represent use on patients who would not normally be readmitted.

(08) FINAL TIME = 100

Units: Month

The final time for the simulation.

(09) Fraction of Discharges with Red Alerts= 0.05

Units: Dmnl [0.01,1]

(10) Individual Interest in Score= MIN(1, Institutional Interest in Score + Perceived Success )

Units: Dmnl

We're assuming people's interest can be driven by institutional interest and by direct evidence of success, with a maximum interest of 1 (meaning the person will always pay attention to the score)

(11) Individual Use Likelihood= INTEG ( Change in Use, 0)

Units: Dmnl

(12) INITIAL TIME = 0

Units: Month

The initial time for the simulation.

(13) Institutional Interest in Score= MIN(1, (Discussion on Rounds + CMS Excess Readmission Rate Penalty) / Attention to Conflicting Priorities)

Units: Dmnl

(14) Likelihood of Red Alert Use= (Number of Trained Individuals / Number of People Involved in Discharges) * Individual Use Likelihood

Units: Dmnl

(15) Normal Readmission Rate= 0.05

Units: Dmnl [0,0.5]

Without use of the tool, this is the readmission rate we would expect

(16) Normal Time to Train= 1

Units: Month [1,10]

(17) Number of People Involved in Discharges= 100

Units: People [0,500]

(18) Number of Trained Individuals= INTEG (Training Flow-Trained Turnover,

0)

Units: People

(19) Perceived Success= Readmission Avoidance Rate * Discharges per Month / Required Evidence

Units: Dmnl

How many people are being helped relative to the number needed to be compelling to care team

(20) Readmission Avoidance Rate= Likelihood of Red Alert Use * Effectiveness of Score Use * Fraction of Discharges with Red Alerts

Units: Dmnl

(21) Readmission Rate= Normal Readmission Rate - Readmission Avoidance Rate

Units: Dmnl

Readmissions fall when patients are given red alert priority

(22) Required Evidence= 100

Units: People/Month [1,100]

How many people per month being prioritized effectively is needed to show clear success to someone using the score

(23) SAVEPER = TIME STEP

Units: Month [0,?]

The frequency with which output is stored.

(24) Score Quality= 1

Units: Dmnl [0,1]

This says what fraction of the patients given red alerts would avoid readmission. It is a baseline value under the conditions that (a) all red alerts are given attention, (b) the red alert fraction is equal to the normal readmission rate, The actual avoided readmissions (see the Readmission Rate and Perceived Success equations) will be lower if red alerts are not being attended to and if the number of red alerts starts to climb relative to actual likely readmissions.

(25) Staff Turnover Rate= 0.01

Units: Dmnl/Month [0,0.1]

(26) TIME STEP = 0.125

Units: Month [0,?]

The time step for the simulation.

(27) Time to Adjust Work Practices= 3

Units: Month [1,12]

(28) Trained Turnover= Staff Turnover Rate * Number of Trained Individuals

Units: People/Month

(29) Training Flow= Institutional Interest in Score * (Untrained People / Normal Time to Train )

Units: People/Month

(30) Untrained People= Number of People Involved in Discharges - Number of Trained Individuals

Units: People
